# New Glass-Ceramics in the System Ca_2_SiO_4_-Ca_3_(PO_4_)_2_—Phase Composition, Microstructure, and Effect on the Cell Viability

**DOI:** 10.3390/ma18163887

**Published:** 2025-08-19

**Authors:** Irena Mihailova, Petya Dimitrova, Georgi Avdeev, Radostina Ivanova, Hristo Georgiev, Milena Nedkova-Shtipska, Ralitsa Teodosieva, Lachezar Radev

**Affiliations:** 1 Department of Silicate Technology, University of Chemical Technology and Metallurgy, 8 Kl. Ohridski Blvd., 1797 Sofia, Bulgaria; 2 Department of Immunology, The Stephan Angeloff Institute of Microbiology, Bulgarian Academy of Sciences, 1113 Sofia, Bulgaria; petya_dimitrova@web.de; 3 Institute of Physical Chemistry “Rostislaw Kaischew”, Bulgarian Academy of Sciences, Akad. G. Bonchev Str., Bl. 11, 1113 Sofia, Bulgaria; g_avdeev@ipc.bas.bg; 4 Department of Inorganic and Electrochemical Production, University of Chemical Technology and Metallurgy, 8 Kl. Ohridski Blvd., 1797 Sofia, Bulgaria; radostina.ivova@abv.bg (R.I.); rteodosieva@uctm.edu (R.T.); l_radev@abv.bg (L.R.); 5 Department of Industrial Safety, University of Chemical Technology and Metallurgy, 8 Kl. Ohridski Blvd., 1797 Sofia, Bulgaria; hristogeorgiev@uctm.edu (H.G.); milenashtipska@uctm.edu (M.N.-S.)

**Keywords:** glass-ceramics, Ca_2_SiO_4_-Ca_3_(PO_4_)_2_ solid solutions, silicocarnotite, 6Ca_2_SiO_4_·Ca_3_(PO_4_) or Ca_15_(PO_4_)_2_(SiO_4_)_6_, α-Ca_2_SiO_4_, cell viability, HL-60 osteoclast-like cells

## Abstract

The CaO-SiO_2_-P_2_O_5_ system is one of the main systems studied aiming for the synthesis of new bioactive materials for bone regeneration. The interest in materials containing calcium-phosphate-silicate phases is determined by their biocompatibility, biodegradability, bioactivity, and osseointegration. The object of the present study is the synthesis by the sol-gel method of biocompatible glass-ceramics in the Ca_2_SiO_4_-Ca_3_(PO_4_)_2_ subsystem with the composition 6Ca_2_SiO_4_·Ca_3_(PO_4_)_2_ = Ca_15_(PO_4_)_2_(SiO_4_)_6_. The phase-structural evolution of the samples was monitored using X-ray diffraction analysis (XRD), Fourier transform infrared spectroscopy (FTIR), scanning electron microscopy (SEM), energy-dispersive spectroscopy (EDS), and surface area analysis. A powder (20–30 µm) glass-ceramic material containing fine crystalline aggregates of dicalcium silicate and plates of silicon-substituted hydroxyapatite was obtained after heat treatment at 700 °C. After heat treatment at 1200 °C, Ca_15_(PO_4_)_2_(SiO_4_)_6_, silicocarnotite Ca_5_(PO_4_)_2_(SiO_4_), and pseudowollastonite CaSiO_3_ were identified by XRD, and the particle size varied between 20 and 70 µm. The compact glass-ceramic obtained at 1400 °C contained Ca_2_SiO_4_-Ca_3_(PO_4_)_2_ solid solutions with an α-Ca_2_SiO_4_ structure as a main crystalline phase. SEM showed the specific morphology of the crystalline phases and illustrated the trend of increasing particle size depending on the synthesis temperature. Effects of the glass-ceramic materials on cell viability of HL-60-derived osteoclast-like cells and on the expression of apoptotic and osteoclast-driven marker suggested that all materials at low concentrations, above 1 µg mL^−1^, are biocompatible, and S-1400 might have a potential application as a scaffold material for bone regeneration.

## 1. Introduction

Bioceramics have attracted increased research attention due to their easy preparation and excellent biocompatibility. The development of different glass-ceramics has provided promising alternatives for replacing part of the skeletal system with material with good osteoconductivity and osteoinductivity [1]. In this context, tricalcium phosphate (Ca_3_(PO_4_)_2_=C_3_P) is one of the most valuable glass-ceramics, based on phosphates, that simulates the mineralogical structure of bone and can act as a bone scaffold for repair and regeneration [2,3]. On the other hand, the incorporation of silica (SiO_2_) into the lattice of C_3_P enhances the bone formation in vivo [4,5,6,7]. Si containing C_3_P has been considered a biodegradable material, which increases the speed of the time-dependent repair, new bone formation, and/or bone regeneration [8,9,10], because the concentration of Si, released into the environment strongly affects protein secretion as well as cell survival and mineralization [11,12]. It is well known that dicalcium silicate (Ca_2_SiO_4_=C_2_S) has been an important constituent in Portland cement and spontaneously develops strength against water [13,14,15]. Moreover, calcium silicate has been widely investigated due to its bioactivity, biodegradability, and biocompatibility [16,17,18,19,20,21,22,23]. Different types of bioceramics, based on C_2_S, have been fabricated for osteogenic and angiogenic responses [24,25], ondogenic and angiogenic differentiation of human cells [26], and bone regeneration [16]. The binary C_2_S–C_3_P system was first studied about 130 years ago, when silicocarnotite (SC) was described as a component of slag [27]. The diagram of phase relations between C_2_S and C_3_P shows the possibility of obtaining C_2_S-C_3_P solid solutions [28]. Taking this into account, Martinez et al. synthesized materials of α-C_3_P doped with a small amount of C_2_S by solid state reaction from the mixture of 95.5 mass % Ca_3_P and 4.5 mass % γ-C_2_S. After thermal treatment, they detected α-C_3_P and SC in the synthesized sample [1]. Later, Martinez et al. identified the presence of monophasic SC by XRD in the sample consisting of 71 mass % C_3_P and 29 mass % C_2_S [29]. α-C_3_P can be doped with 1.5 mass % or 3.0 mass % C_2_S in the C_2_S-C_3_P system by solid state reaction, and the obtained materials are composed of a single phase of α-C_3_P or solid solution of C_2_S in α-C_3_P for the doped α-C_3_P [30]. $\alpha_{L}^{\prime }$-C_2_S doped with phosphorus in the subsystem C_2_S-7CaO.P_2_O_5_.2SiO_2_ has been used for the preparation of porous scaffolds by the burnout technique [31], and $\alpha_{L}^{\prime }$-C_2_S is present in the prepared sample after thermal treatment [31]. Lugo et al. have identified two intermediate compounds in the C_2_S-C_3_P system: an incongruently melting compound 2C_2_S.C_3_P with chemical formula Ca_7_(PO_4_)_2_(SiO_2_)_2_, known as Nurse’s A-phase [32], and the second phase of SC [33]. On the other hand, the system of Nurse’s A-phase-SC was conducted as a preliminary step toward obtaining new biomaterials with a controlled microstructure, as noted by Ros-Tarraga et al. [34] and Rubio et al. [35]. Lugo et al. synthesized a single A-phase material with a molar ratio of C_2_S and C_3_P of 2:1 [32]. However, depending on the P_2_O_5_ in the CaO-SiO_2_-P_2_O_5_ system, three phases could be formed after thermal treatment at 1250 °C for 1 h: larnite (L), Ca_14.92_(PO_4_)_2.35_(SiO_4_)_5.65_, and 5CaO.SiO_2_.P_2_O_5_ [15]. Radev et al. prepared glass-ceramics in the C_2_S-C_3_P system via the poly-step sol-gel method, at a different molar ratio [36,37,38,39]. They reported the identification of Ca_15_(PO_4_)_2_(SiO_4_)_6_ among the main phases in the synthesized glass-ceramics. The in vitro bioactivity of these glass-ceramics in a simulated body fluid environment has been established [36,37,38,39]. The crystal structure of Ca_15_(PO_4_)_2_(SiO_4_)_6_ was first described by Saalfeld and Klaska as a structure derivative of α’-C_2_S, and the chemical composition of the crystals has also been experimentally determined [40]. According to Widmer et al., Ca_15_(PO_4_)_2_(SiO_4_)_6_ or 6C_2_S.C_3_P is a distinct phase in the C_2_S-C_3_P subsystem [41]. The reason for excluding it in the phase diagram is its structural similarity to $\alpha_{H}^{\prime }$-Ca_2_SiO_4_ and $\alpha_{L}^{\prime }$-Ca_2_SiO_4_ and the difficulty in distinguishing these three phases in multiphase mixtures by means of powder XRD [41].

The object of the present study is to synthesize by the sol-gel method glass-ceramics in the C_2_S-C_3_P system with the composition 6C_2_S.C_3_P and to study them. We aim to follow the structural and phase evolution of the glass-ceramics depending on the synthesis temperature and to determine the corresponding influence on cell viability. This would provide the prospective guidelines for their application as biomaterials for bone regeneration.

## 2. Materials and Methods

### 2.1. Materials

All chemicals used for the modified sol-gel synthesis were reagent grade: >99% tetraethoxysilane, TEOS (C_2_H_5_O)_4_Si, Alfa Aesar, Heysham, Lancashire, UK), 98% calcium oxide (CaO, Valerus, Sofia, Bulgaria), 85% phosphoric acid (H_3_PO_4_, Valerus, Sofia, Bulgaria), 37% hydrochloric acid (HCl, Merck, Darmstadt, Germany), 25% ammonium hydroxide (NH_4_(OH), Valerus, Sofia, Bulgaria), 99.99% Ethanol (C_2_H_5_OH, Valerus, Sofia, Bulgaria), and deionized water served as solvents in this study.

### 2.2. Methods

#### 2.2.1. Synthesis of the Glass-Ceramics

The nominal chemical composition of the prepared glass-ceramics is described as 6Ca_2_SiO_4_·Ca_3_(PO_4_)_2_ or 76.91 mass % Ca_2_SiO_4_—23.09 mass % Ca_3_(PO_4_)_2_. The glass-ceramics were synthesized by a poly-step sol-gel method, similar to that described in our previous studies [39]. In addition to the well-known methods for the synthesis of biomaterials, solid-state, sol-gel, hydrothermal, precipitative, etc., new alternative synthesis methods such as spark plasma sintering–reactive synthesis [41,42,43,44] are also proposed to meet certain requirements for structural and mechanical properties of the materials. We use sol-gel because this method is a purely chemical process during which various irreversible reactions occur, and the product is obtained. Furthermore, the sol-gel method is capable of producing highly pure and uniform materials compared to other methods, which is very advantageous [45]. Initially, two solutions were prepared, the first corresponding to the composition of calcium disilicate—(sol I) and the second corresponding to tricalcium phosphate—(sol II). Calcium disilicate sol I was obtained by appropriate amounts of Ca(OH)_2_ and SiO_2_. Ca(OH)_2_ was prepared by dissolution of CaO in deionized water under stirring. Silicate sol was produced by hydrolysis of TEOS in the presence of C_2_H_5_OH, H_2_O, and HCl in a molar ratio of TEOS:C_2_H_5_OH:H_2_O:HCl = 1:1:4:0.01. After stirring the silicate and Ca(OH)_2_ solutions for 1 h, they were mixed. The sol II contains CaO and H_3_PO_4_ in a ratio corresponding to the stoichiometry of Ca_3_(PO_4_)_2_. The required amount of H_3_PO_4_ was added to the Ca(OH)_2_ solution obtained from CaO and deionized water. NH_4_OH (25%) was used to obtain pH = 10–11 of mixed calcium phosphate (CP) solution. The sol I was added from the burette to the sol II. The mixture was stirred for 12 h. The obtained mixed sol was dried at 120 °C for 12 h and thermally treated at a temperature of 700 °C for 3 h. The resulting sample is designated S-700. This temperature was determined according to the data obtained from the thermal analysis. The STA PT1600 TG-DTA/DSC (STA—Simultaneous Thermal Analysis) apparatus, manufactured by LINSEIS Messgeräte GmbH, Selb, Germany, was used to conduct thermal analysis (Differential Thermal Analysis (DTA) and Thermogravimetry (TG)) of the dry gel. The analysis conditions were air environment and a sample heating rate 10°/min. The dry gel was homogenized in an agate mortar and then was heated in a corundum crucible. Two other glass-ceramic samples were obtained by additional heat treatment for 2 h in corundum crucibles at 1200 or 1400 °C, respectively. They are designated S-1200 and S-1400. The higher heating temperatures were chosen based on literature data on the polymorphic transitions of C_2_S. After heating, the samples were allowed to cool in the furnace.

#### 2.2.2. Characterization of the Glass-Ceramics

The chemical composition of the synthesized samples was experimentally determined by means of inductively coupled plasma optic emission spectrometry. A High Dispersion ICP-OES Prodigy spectrometer from Teledyne Leeman Labs (Hudson, NY, USA) was used. Powder X-ray diffraction (XRD) analysis was applied for crystalline phase identification. An X-ray diffractometer Empyrean (PANalytical, Malvern Panalytical, Almelo, Netherlands) was used. The conditions for conducting the analysis were as follows: CuKα radiation, 2θ range from 5° to 90°, step size 0.05°, and counting time 74 s. The powder diffraction files (PDF) # 01-084-1997; # 01-083-1494; # 00-040-0393; and # 01-086-0401 from the database JCPDS-International Centre for Diffraction Data were used together with Match! 2 software. Rietveld analysis was performed for the quantification of the crystalline phases and the determination of the unit cell parameters. For Rietveld refinements, HighScore Plus 4.0 software was used. The Fourier transform infrared spectroscopy (FTIR) has also been applied for phase analysis. The pressed-pellet technique in KBr was used for sample preparation. The FTIR measurements were performed with a spectrometer Varian 600-IR series (Varian, Melbourne, Australia), in the frequency range from 4000 to 400 cm^−1^. Observations of the microstructure of the synthesized glass-ceramic samples were carried out by scanning electron microscopy (SEM). The surfaces of the prepared samples were observed using SEM EVO 10 (Carl Zeiss GmbH, Jena and Oberkochen, Germany) equipment with energy dispersive spectroscopy (EDS) facilities—Oxford Instruments, Xplore 30, Oxford, UK. Optical microscopic observations were performed too. The samples were observed in reflected light with a Stemi 305 stereomicroscope (Carl Zeiss IQS Deutschland GmbH, Oberkochen, Germany) equipped with an Axiocam 208 camera (Zeiss, Carl Zeiss IQS Deutschland GmbH, Oberkochen, Germany). They were further examined in immersion in transmitted light with an Axioscope 5 polarization microscope (Carl Zeiss Microscopy GmbH, Jena, Germany) with an Axiocam 208 camera (Zeiss, Carl Zeiss IQS Deutschland GmbH, Oberkochen, Germany). To determine the adsorption-texture parameters of the synthesized samples, a surface area and pore surface area analyzer, model NOVA 1200e, Quantachrome (Quantachrome Instruments, Boynton Beach, FL, USA) was used. Specific surface area was determined by low temperature (77.4 K) nitrogen adsorption using the Brunauer–Emmett–Teller (BET) method. Gurvitch’s rule was applied to determine total pore volume. Average pore diameter, micropore volume, specific surface area associated with micropores, and external surface area were evaluated according to the V-t method.

#### 2.2.3. Materials and Methods for Assessing Cell Viability

##### Cell Line and Culturing

The HL-60 cell line (#CCL-240, ATCC; Washington, DC, NW, USA) was a gift from Prof. Giovanni Bernardini from the University of La Sapienza (Rome, Italy). The line was cryopreserved in tubes (#430659, Corning, Wiesbaden, Germany) with 1 mL of 20% DMSO, 10% fetal bovine serum (FBS; #P40-37100, PAN Biotech, Aidenbach, Germany), Roswell Park Memorial Institute 1640 (RPMI) with L-Glutamine, and NaHCO_3_ (#RPMI-A, Capricorn Scientific, Dreihausen, Germany) in liquid nitrogen (Taylor-Wharton, Livermore, CA, USA).

After thawing, HL-60 cells were washed by centrifuging at 200× *g* with pre-warmed 10% FBS/RPMI medium supplemented with amino acids (#R7131, Sigma-Aldrich, Darmstadt, Germany) and antibiotics (#R8758, Sigma-Aldrich, Germany). The cells were seeded at concentration 1 × 10^6^ mL^−1^ in T25 flasks (#83.3910.002, Sarstedt, Germany) and incubated at 37 °C, 5% CO_2_ (Memmert incubator, Schwabach, Germany). After 48 h exponential growth, the cells were collected and treated with vitamin D3 (VitD3) (#5009360010, Merck, Darmsrtadt, Germany) at a concentration of 10^−10^ M to obtain osteoclast-like morphology as described by Yoneda et al., 1991 [46]. The vehicle of 0.01% ethanol in Dulbecco’s phosphate-buffered saline without Ca^2+^ and Mg^2+^ (D-PBS; pH = 7.4; #PBS-1A, Capricorn Scientific, Dreihausen, Germany) was used as a control.

##### Wright Cell Staining

HL-60 osteoclast-cells (1 × 10^5^ cells/mL^−1^) were seeded on a gelatin-pre-coated glass slide (100 µL/slide). The cells were allowed to interact with ceramic materials added at concentrations of 1 and 5 mg mL^−1^ for 20 min at room temperature. Then the cells were fixed with 70% methanol/D-PBS for 10 min at room temperature and stained with Wright stain for 5 min (0.5% solution; Medical Technics Engineering Ltd., Sofia, Bulgaria). The slides were gently washed 3 times with 200 µL/slide dH_2_O and allowed to dry. Then they were observed under a light microscope (Boeco^TM^ BM180, Boeco, Hamburg, Germany) at 20× or 40× magnifications by two independent observers in a blinded manner. Images were captured at 40× magnification by a digital camera (model Nikon Coolpix 8400, Nikon Instruments, Melville, NY, USA).

##### MTT Assay for Cell Growth

A Colorimetric MTT kit (#CT01, Merck KGaA, Darmstadt, Germany) was used to evaluate cell vitality. The assay was based on metabolization by live cells of the yellow tetrazolium salt—(3-(4,5-dimethylthiazol-2-yl)-2,5-diphenyl tetrazolium bromide) (MTT)—and accumulation of the purple formazan crystals in the cytoplasm. The method was performed according to the manufacturer’s protocol and as described by Dimitrova et al., 2025 [47]. Briefly, HL-60 cells (1 × 10^5^ cells mL^−1^, 100 µL/well) were seeded in 96-well plates (#Z707910, TPP, Sigma-Aldrich, Germany) and cultured in the presence of the ceramic materials at concentrations of from 0.3 or 1.5 to 100 µg mL^−1^ (100 µL/well). Positive controls with growth medium only or with the vehicle 0.01% methanol/D-PBS were performed. After 48 h of culture, 0.01 mL MTT/D-PBS solution was added (100 µL/well) and incubated for 4 h at 37 °C to allow the metabolization of the MTT and the formation of the formazan black crystals in vital cells. The formazan crystals were then dissolved by 0.04 N HCl in 70% methanol (100 µL/well) The absorbance was measured within 20 min using an ELISA plate reader (BioTek EL 800, Winooski, VT, USA) with a wavelength of 590 nm and a reference wavelength of 630 nm. The cell vitality was calculated as a percentage (%) of the control. The dose-response logarithmic curves for the cell growth inhibition were plotted, and the log_10_ inhibitory concentration (IC)_50_ was determined by a four-parameter logistic regression model of the AAT Bioquest, Inc., Pleasanton, CA, USA (Quest Graph^TM^ IC50 Calculator, retrieved from https://www.aatbio.com/tools/ic50-calculator (accessed on 19 May 2025). The value for each sample was represented as the logIC_50_ according to the paper of Srinivasan and Lloyd, 2024 [48].

##### Flow Cytometry for Evaluation of Surface TRAIL

Cultured HL-60 cells with osteoclast phenotype (1 × 10^5^ mL^−1^) were harvested, washed 3 times with 0.5 mL D-PBS, resuspended in 2% BSA (bovine serum albumin)/D-PBS (100 µL/tube), and stained with either antibody against human TRAIL (0.1 mg/mL, clone RIK-2, # 308205, Biolegend, London, UK) labeled with phycoerythrin (PE) or with isotype control monoclonal mouse IgG1conjugated with PE (clone MOPC-21, # 400111, Biolegend, London, UK). After incubation for 20 min, the cells were washed with 2%BSA/D-PBS and finally resuspended in 300 µL/tube D-PBS and then subjected to flow cytometry collecting 20,000 events/tube using BSR II flow cytometer (BD Biosciences, San Jose, CA, USA) with DIVA 6.0 software.

### 2.3. Statistical Analysis

Standard deviations (SDs) were calculated for the parameters where applicable. The ANOVA test was used to compare the data. The *p*-values calculated for *p* < 0.05 (5%) were considered statistically significant.

## 3. Results and Discussion

As a result of the syntheses, three materials were obtained. Sample S-700 was in the form of a fine powder. The samples S-1200 and S-1400 obtained at temperatures of 1200 and 1400 °C, respectively, are shown in Figure S2. The powder material, which was placed in the alumina crucible, underwent varying degrees of compaction (shrinkage) and formed cylindrical bodies. Sample S-1200 was not sintered and monolithic but was easily broken and pulverized. Sample C-1400 was a relatively dense body with a micro-grained structure and no visible macroscopic pores. In Figure S3, micrographs of the cut surface of sample S-1400 and of the chipped surface of sample S-1200 are shown. Although sample S-1400 was macroscopically dense, optical microscopic observations revealed pores with sizes of 0.1–0.3 mm and single pores with a size of ≈0.5 mm. The sample was monolithic and could be cut, but its mechanical properties were not comparable to those of bone. The Mohs hardness was determined to be between 2.5 and 3. Sample S-1200 was highly porous, had no mechanical strength, and could be described as powdery. Transmission light microscopy images of the three samples in an immersion liquid are shown in Figure S4. Samples S-700 and S-1200 were not subjected to additional grinding. They illustrate that the predominant particle size in the composition of sample S-700 is 20–30 µm, while in S-1200, the particle size varies between 20 and 70 µm. The images in cross-polarized light (XPL) mode show interference colors. Therefore, the presence of optically anisotropic crystalline phases was found in the three samples.

### 3.1. Chemical Composition of Glass-Ceramics

The experimental results for the chemical composition of the synthesized glass-ceramic sample S-1200 are shown in Table 1 and compared with the nominal chemical composition. The experimental composition is close to the nominal one. The differences are in the order of the uncertainty values. The additional oxides in the experimental composition are introduced with the used starting materials. The impurity of alumina is probably due to the thermal treatments of the materials in corundum crucibles.

### 3.2. X-Ray Diffraction Analysis (XRD) of the Glass-Ceramics Synthesized After Heating at Different Tempratures

**Figure 1:**
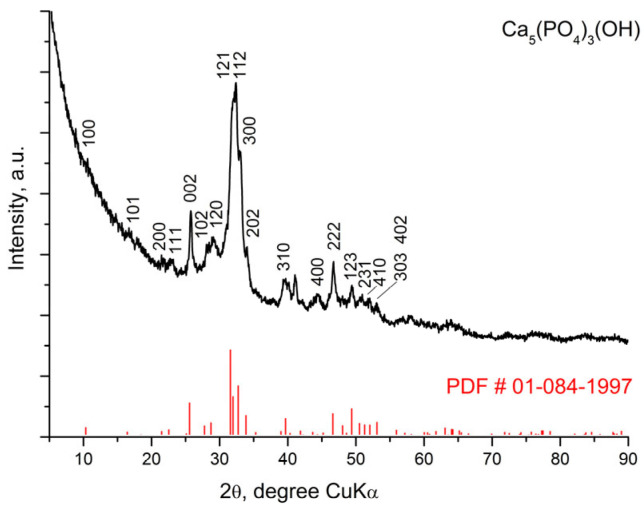
XRD pattern of glass-ceramic sample S-700.

**Figure 2:**
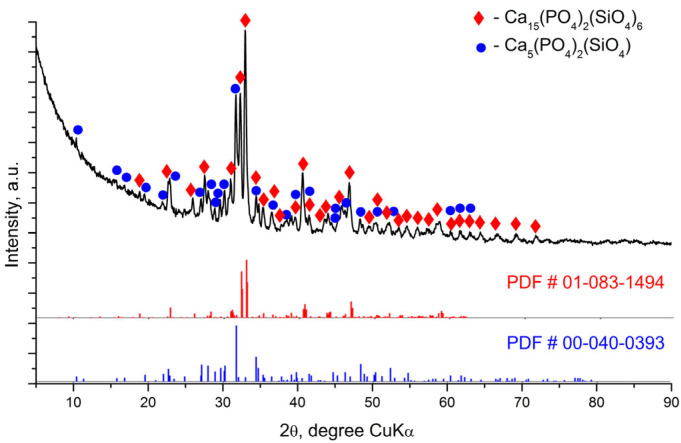
XRD pattern of glass-ceramic sample S-1200.

**Figure 3:**
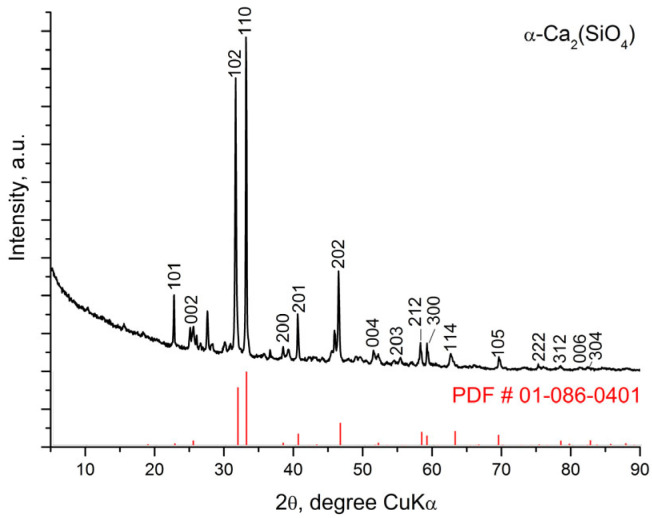
XRD pattern of glass-ceramic sample S-1400.

Figure 1, Figure 2 and Figure 3 represent the X-ray diffraction data of the glass-ceramic samples.

The X-ray diffraction pattern of the sample S-700 (Figure 1) indicates the presence of an amorphous halo as well as diffraction peaks. When comparing the experimental X-ray diffraction data of the sample with a PDF # 01-084-1997 for apatite (Ca_5_ (PO_4_)_3_(OH) hexagonal symmetry, SG *P*6_3_/*m* (176)), good agreement was found. Therefore, hydroxyapatite crystals were formed during the sol-gel synthesis. A similar phase formation, namely the formation of a hydroxyapatite phase, has been registered for other compositions in the CaO-SiO_2_-P_2_O_5_ system in our previous syntheses using the described modified sol-gel method. It is likely that apatite was formed when mixing 85% H_3_PO_4_ with the aqueous suspension containing CaO in a basic environment at pH 10–11. The required pH value was maintained by adding ammonia solution (25% NH_3_).

More precise data on the phase composition of sample S-700 were obtained by Rietveld analysis. The analysis data confirmed the presence of hydroxyapatite in the sample, but the presence of another crystalline phase, β-Ca_2_(SiO_4_), was also found. Information on weight fractions and crystallographic characteristics of the phases is presented in Table 2.

Figure 2 shows the experimental XRD data for the S-1200 sample, indicating the peaks of the main crystalline phases Ca_15_(PO_4_)_2_(SiO_4_)_6_ and silicocarnotite. The unit cell parameters of the crystal phases and the quantitative ratio between them were determined by Rietveld analysis and are presented in Table 3. Figure 3 shows that the main diffraction peaks in the X-ray diffraction pattern of sample S-1400 correspond to the crystalline structure of the high-temperature polymorph of dicalcium silicate α-Ca_2_SiO_4_, which has hexagonal symmetry SG *P*6_3_/*mmc* (194) according to # 01-086-0401. The accompanying crystalline phases are Ca_15_(PO_4_)_2_(SiO_4_)_6_ and Pseudowollastonite CaSiO_3_ (Table 4).

The results of the X-ray diffraction analysis provide the following basic information about the phase composition of the glass-ceramic samples depending on the applied thermal treatment. The S-700 sample contains two crystalline phases—C_2_S phase and hydroxyapatite. Three new crystalline phases were identified after heating at 1200 °C for 2 h. One of them structurally corresponds to Ca_15_(PO_4_)_2_(SiO_4_)_6_ [40] and the others to silicocarnotite Ca_5_(PO_4_)_2_(SiO_4_) and pseudowollastonite CaSiO_3_. The crystalline phase present in the highest amount is Ca_15_(PO_4_)_2_(SiO_4_)_6_, followed by silicocarnotite and pseudowollastonite. Hydroxyapatite is also recorded in small amounts. The crystalline structure of Ca_15_(PO_4_)_2_(SiO_4_)_6_ was described by Saalfeld and Klaska [40]. This phase can be considered as a high-temperature polymorph of C_2_S (α’-C_2_S) stabilized by C_3_S. The crystalline phases in glass-ceramic S-1200 can be considered as being formed as a result of phase transformation in S-700; namely, silicocarnotite was formed by the inclusion of more silicate groups in the hydroxyapatite structure, and Ca_15_(PO_4_)_2_(SiO_4_)_6_ was formed during the transformation of C_2_S. The samples obtained after heating at 1400 °C for 2 h show a structural difference. It is likely that a solid solution between Ca_2_SiO_4_ and Ca_3_(PO_4_)_2_ is formed as a main crystalline phase whose structure corresponds to the high-temperature modification of calcium disilicate α-Ca_2_SiO_4_ with hexagonal symmetry.

### 3.3. Fourier Transform Infrared Spectroscopy (FTIR) of the Glass-Ceramics Synthesized After Heating at Different Temperatures

**Figure 4:**
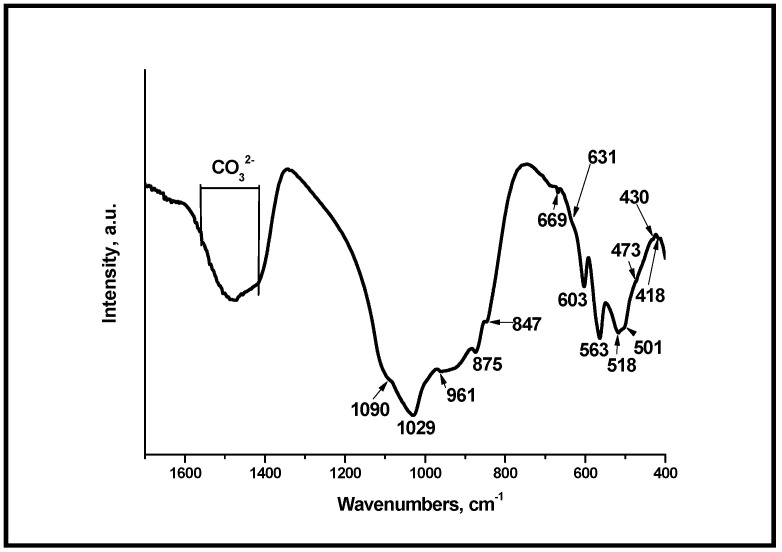
FTIR spectra of the glass-ceramic sample S-700.

It is well known that the PO_4_^3−^ groups in hydroxyapatite present four main vibration domains: ν_1_ (around 950 cm^−1^), ν_2_ (400–470 cm^−1^), ν_3_ (1000–1150 cm^−1^), and ν_4_ (500–620 cm^−1^) [49]. In our case, the bands positioned at 418, 430, and 473 cm^−1^ (Figure 4) can be ascribed to ν_2_ PO_4_^3−^ [50]. Those at 501, 518, 563, and 603 cm^−1^ can be ascribed to ν_4_ PO_4_^3−^ [49,51], and a peak at 669 cm^−1^ can be related to ν_4_ PO_4_^3−^ [52]. The peaks centered at 418, 430, and 473 cm^−1^ could be related to the ν_2_ PO_4_^3−^ [51,53]. The peaks at 961, 1029, and 1090 cm^−1^ could be associated with the vibrations ν_1_ and ν_3_ PO_4_^3−^ [51,53]. In addition, the peaks with low intensity at 847 and 875 cm^−1^ can be related to ν_2_ CO_3_^2−^ in accordance with [54]. The peak centered at 631 cm^−1^ can be related to OH^−^ in HA structures, in accordance with [55]. On the other hand, the absorption peaks, centered at 1420–1560 cm^−1^ could be ascribed to the presence of CO_3_^2−^ ions in HA structure, i.e., the carbonate contacting HA (CO_3_HA) can be formed after thermal treatment at 700 °C of the synthesized sample [56].

**Figure 5:**
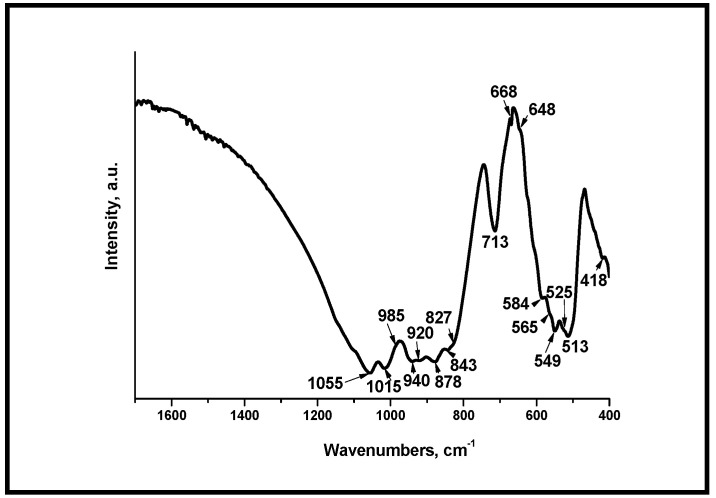
FTIR spectra of the glass-ceramic sample S-1200.

**Figure 6:**
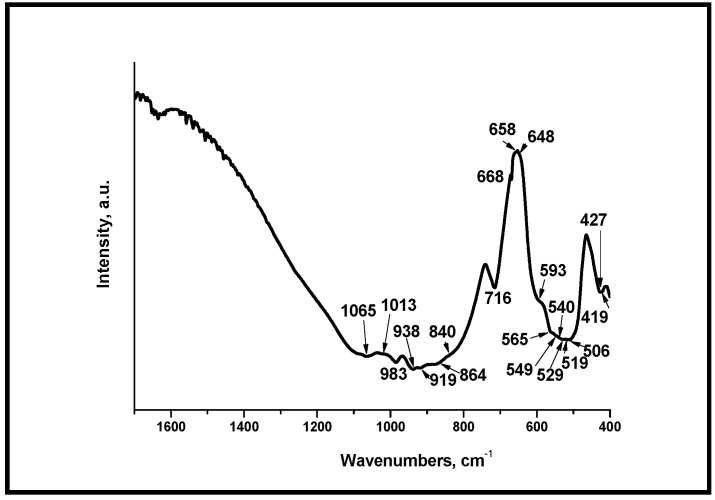
FTIR spectra of the glass-ceramic sample S-1400.

Figure 5 and Figure 6 present the FTIR spectra of the samples, after thermal treatment at different temperatures. From the FTIR data presented in Figure 5, we can conclude that the absorption bands of silicate groups were clearly evident. The band at 985 cm^−1^ was assigned to the Si-O-Si symmetric stretching and those at 940 and 920 cm^−1^ to the Si-O symmetric stretching vibrations [38]. The well visible peaks, positioned at 549 and 513 cm^−1^, could be related to the Si-O-Si vibrational mode of bending [38,57]. The peak centered at 878 cm^−1^ was also detected. It can be assigned to the presence of SiO_4_^4−^ in the prepared sample [36]. The peaks posited at 648, 668, and 713 cm^−1^ could be related to Si-O bending vibrations [38]. Furthermore, the absorption bands at 1015 and 1055 cm^−1^ can be assigned to the vibration of the Si-O-Si bond [36]. On the other hand, the peaks at 418, 549, 565, and 584 cm^−1^ could be related to ν_3_ PO_4_^3−^ and ν_4_ PO_4_^3−^, in accordance with [36,38,57]. The bands centered at 920, 940, 1015, and 1055 cm^−1^ can also be related to the ν_1_ PO_4_^3−^ and ν_3_ PO_4_^3−^ in the as-prepared and in thermally treated samples [36,38,57]. The FTIR results strongly indicated that the presence of silicocarnotite in the sample was supported by the identified bands at 418 (419), 513, 549, 565, 668, 920, 940, and 1055 cm^−1^. These data were relevant to other studies that identified silicocarnotite in the ceramic materials by FTIR [38,57]. In addition, the interpretation of the FTIR data is in agreement with the determined phase composition of the samples.

Figure 6 shows FTIR spectra of the synthesized sample, after heat treatment at 1400 °C for 2 h. In the presented spectrum, the absorption bands for phosphate and silicate units are also clearly visible as well as in Figure 5. The band positioned at 983 cm^−1^ was assigned to the Si-O-Si asymmetric stretching, the bands at 938 and 919 cm^−1^ to the Si-O symmetric stretching, and the bands, centered at 549 and 519 cm^−1^ to the Si-O-Si bending vibration [36,38]. The bands at 549, 648, 668, 1013, and 1065 cm^−1^ can be assigned to the presence of ν_3_ or ν_4_ SiO_4_^4−^ [36,38,57]. The band at 716 cm^−1^ is the band due to Si-O bending vibrations, and the bands centered at 419, 427, 506, and 1013 cm^−1^ can be associated with ν_3_ PO_4_^3−^ [36,57,58,59]. In addition, the bands at 549, 565, and 593 cm^−1^ can be attributed to ν_4_ PO_4_^3−^ [36,38,57].

The interpretation of the FTIR data is in agreement with the determined phase composition of the samples.

### 3.4. Analyses of the Glass-Ceramic Materials by Scanning Electron Microscopy (SEM) and Energy-Dispersive Spectroscopy (EDS)

**Figure 7:**
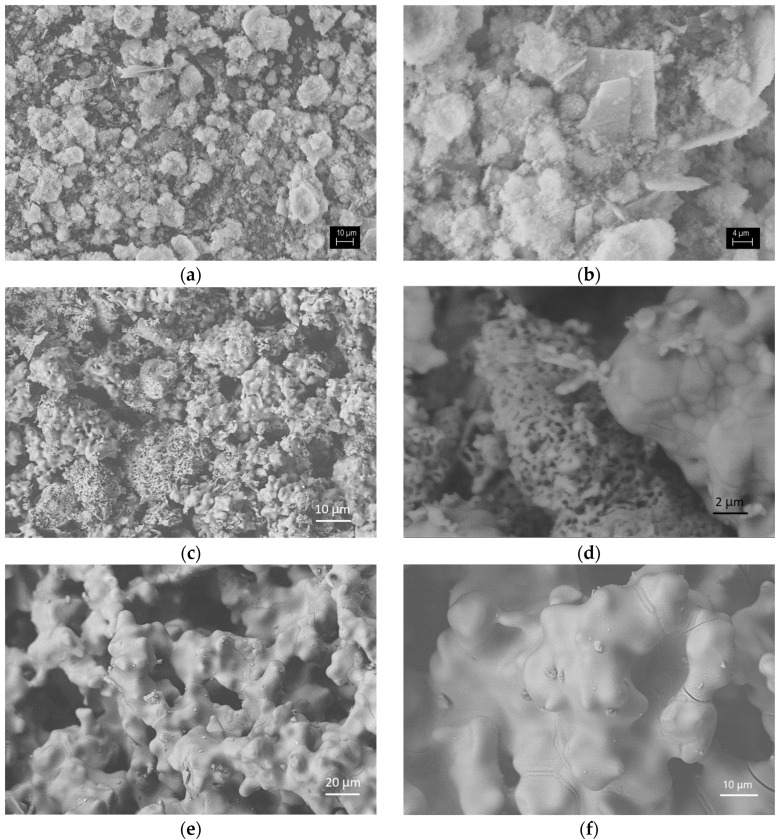
Microstructure of the glass-ceramics samples: (**a**) S-700, magnification 513×; (**b**) S-700, 1500×; (**c**) S-1200, 1000×; (**d**) S-1200, 5000×; (**e**) S-1400, 1000×; (**f**) S-1400, 3000×.

**Figure 8:**
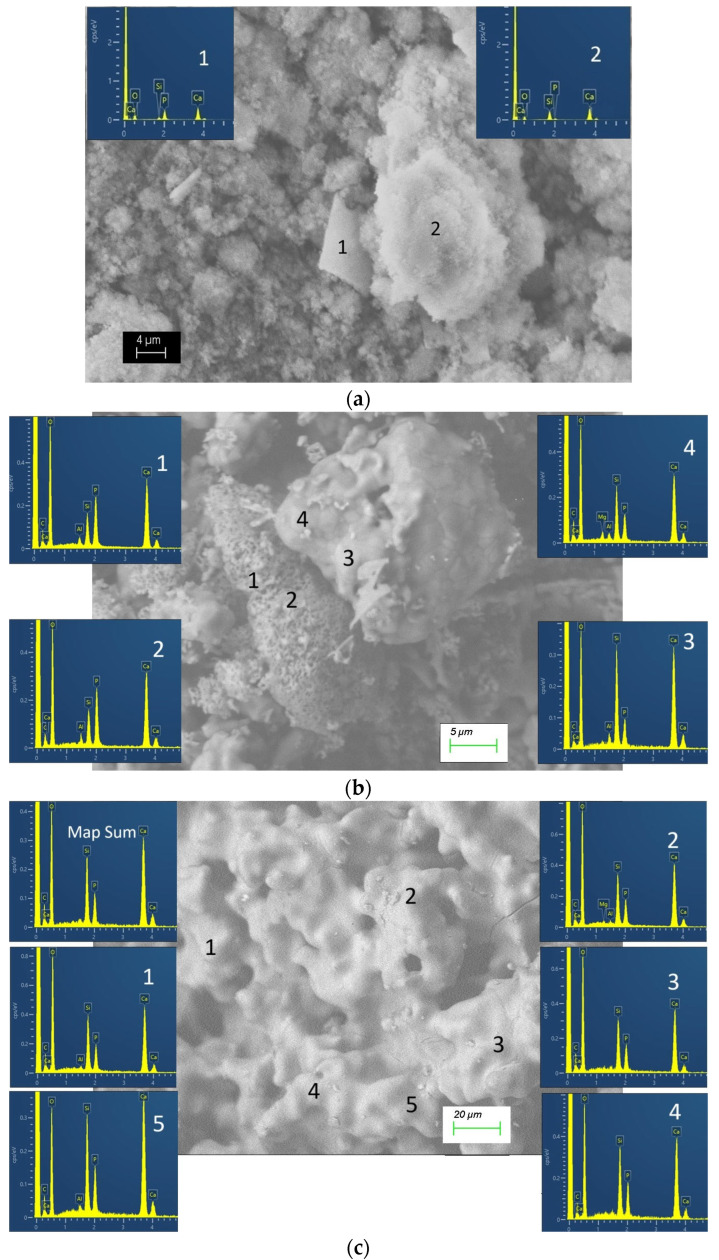
EDS data for the glass-ceramic samples: (**a**) S-700; (**b**) S-1200; (**c**) S-1400. The numbers on the microphotographs indicate the points of the corresponding EDS analyses.

In sample S-700, the presence of aggregates of particles with sizes smaller than 1 μm was found (Figure 7a,b). In turn, the aggregates reach 10–20 μm. In addition to fine particles, rectangular plates with a thickness of less than 1 μm and a wall length reaching 10–20 μm were found in the sample. The chemical composition of the fine-grained aggregates showed a composition close to dicalcium silicate, and that of the plates is close to the calcium phosphate phase (hydroxyapatite) (Figure 8a). In all EDS analyses, Ca, P, Si, and O were found. The detection of carbon is in agreement with the presence of carbonates according to the FTIR data.

Microscopic observations of sample S-1200 allowed us to distinguish two types of crystal aggregates—dense and porous ones (Figure 7c,d). The dense aggregates were sintered with a polygonal structure and crystal sizes of 1–5 μm. In their chemical composition, the Si/P atomic ratio was between 1.73 and 3.59 (Figure 8b). Compositions that corresponded well to the theoretical composition Ca_15_ (SiO_4_)_6_(PO_4_)_2_ have been established, too. The “porous” aggregates were made up of particles with a submicron size. Pores with a size of less than 1 μm were visible too. In the chemical composition of these areas, phosphorus prevailed over silicon. The Si/P ratio was close to 1/2, in accordance with that of silicocarnotite (Figure 8b). The sample obtained after thermal treatment at the highest temperature S-1400 had larger particles (Figure 7e,f). They were irregular in shape and rounded in outline, and their dimensions were over 10–15 μm. The particles with complex morphology appear to be the result of the bonding of several smaller particles. The chemical composition of the formed phases in sample S-1400 allowed them to be determined as solid solutions between C_2_S and C_3_P with atomic ratios Si/P in the range 1.67–1.84 (Figure 8c). The complex polyphase composition of S-1200 and S-1400 is also responsible for the wide range in which the Si/P ratio varies in these samples.

The SEM and EDS analyses performed allowed the structural characteristics of the glass-ceramics and the corresponding chemical composition of the phases to be described. In the samples S-700 and S-1200, the phases with different compositions were clearly distinguished by their morphology. In the chemical composition of all the studied phases in these samples, Ca, Si, and P were found. The main phases were distinguished by the Si/P ratio too. Some of the phases can be considered as calcium phosphates substituted by silicon. For example, in the sample S-700, silicon substituted hydroxyapatite was found. In addition, silicocarnotite is a calcium silico phosphate, the chemical composition of which can be considered hydroxyapatite, in which SiO_4_ replaces one of the PO_4_ groups. On the other hand, the other phase in S-700 has a predominantly calcium silicate composition. C_2_S also prevailed in the phase identified by XRD as Ca_15_(PO_4_)_2_(SiO_4_)_6_. The variations in the chemical composition indicated rather the formation of solid solutions in the C_2_S-C_3_P system with a structure corresponding to Ca_15_(PO_4_)_2_(SiO_4_)_6_. In the sample obtained after heating at the highest temperature, a solid solution was again present, but with an α-C_2_S structure. Despite the variations in the chemical composition at the micro level in this sample, in all the established compositions, silicon prevailed over phosphorus.

The morphologies of the crystalline phases, which structurally correspond to high-temperature polymorphs of C_2_S in samples C-1200 and C-1400, are similar to those published in [60] for β-C_2_S phases doped with 0.5 mass % phosphorus and obtained at similar temperatures. The studies conducted using XRD, FTIR, SEM, and EDS allowed tracing the structural evolution of the samples depending on the maximum temperature applied during synthesis. They showed that the three samples, the subject of the present study, were significantly different in their phase composition and microstructure.

### 3.5. Adsorption-Texture Parameters

The glass-ceramic samples were studied with respect to their low-temperature nitrogen adsorption. Specific surface area by the Brunauer–Emmett–Teller (BET) method and full adsorption isotherm with micropore (below 2 nm) and mesopore (2–50 nm) size distribution were determined. The following parameters were assigned to the obtained samples: S_BET_ (m^2^ g^−1^)—specific surface area by BET; V_total_ (cm^3^ g^−1^)—total pore volume; D_average_ (nm)—average pore diameter; V_mi_ (cm^3^ g^−1^)—micropore volume; S_mi_ (m^2^ g^−1^)—specific surface area associated with micropores; S_ext_ (m^2^ g^−1^)—external surface area. The results are shown in Table 5.

### 3.6. Effect of the Glass-Ceramic Materials on Cell Viability

**Figure 9:**
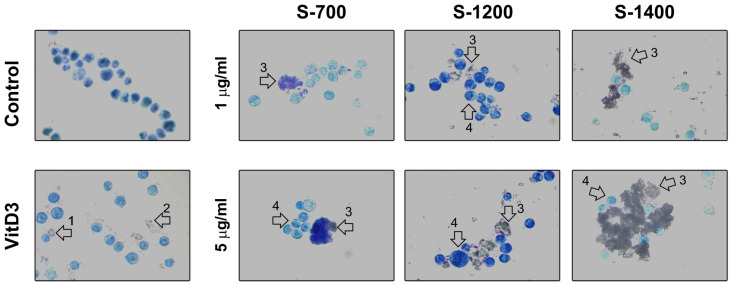
Interaction between the cells and ceramic materials. The cells were treated with VitD3 or vehicle (0.01% ethanol/D-PBS/) for 48 h. Then the cells were washed and incubated with the ceramic samples for 20 min, Wright stained, and subjected to light microscopy. The photomicrographs were taken at magnification 40×. Arrows show 1—apoptotic cells, 2—degranulation, 3—ceramic materials, 4—cells.

**Figure 10:**
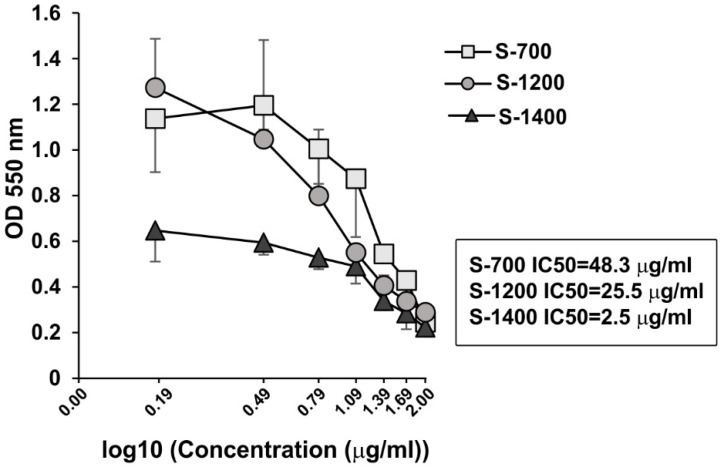
Dose-dependent effect of S-700, S-1200, and S-1400 on cellular viability of osteoclast-like HL-60 cells. HL-60 cells were pre-treated with VitD3 (10^−10^ M) or the vehicle for 48 h. After washing, the cells were cultured in the presence or absence of the materials at concentrations from 1.5 to 100 µg mL^−1^, and cell viability was evaluated by MTT assays after 48 h. Data represent mean ± SD of three repeats/sample of three experiments (*n* = 9). IC_50_ denotes concentrations of the materials that induce 50% inhibition of cellular viability and was calculated using AAT Bioquest Software (https://www.aatbio.com/tools/ic50-calculator (accessed on 19 May 2025).) (Pleasanton, CA, USA).

The differentiation of promyelocyte HL-60 cell line towards an osteoclast-like phenotype has been described by Yoneda et al. [46]. The cells were treated with vitamin D3 (VitD3) at the concentration 10^−10^ M for 48 h. Some morphological and functional characteristics of osteoclasts have been observed, including the presence of tartrate-resistant acid phosphatase activity and the capacity to adhere and resorb bone and to respond to calcitonin [46]. Yoneda et al. named the population HL-60-osteoclast-like cells [46]. In our experiments, the apoptotic (Figure 9, arrow 1) and degranulating cells (Figure 9, arrow 2) have been detected as a result of VitD3-induced cell differentiation and stimulation. The incubation of the ceramic materials with VitD3-treated cells for 20 min resulted in the formation of clusters between the ceramic particles (Figure 9, arrow 3) and the cells (Figure 9, arrow 4). HL-60-osteoclast-like cells clustered around the surface of S-700 and S-1200 particles (Figure 10) but they were distributed within the S-1400 aggregates (Figure 9).

The observed S-1400 aggregates were bigger than those of S-700 and S-1200 at a concentration of 5 µg mL^−1^ (Figure 9). This experiment was performed for a short-time incubation, only 20 min, but it demonstrated a distinct ability of the cells to interact with the ceramic samples. For example, we found that S-700 formed aggregates reaching 10–20 μm (Figure 7a,b) and showed the highest specific surface area (Table 3) that might provide a surface and probably stability for cell adhesion (Figure 9). Indeed, cells interacting with the surface of the material were detected (Figure 9). The sample S-1200 showed two types of crystal aggregates—dense and porous (Figure 7c,d). The dense aggregates were with a polygonal structure, while the “porous” aggregates formed pores with a size of less than 1 μm (Figure 7c,d).

We suggest that the cells were able to interact with the dense and porous crystal aggregates at both concentrations (1 or 5 µg mL^−1^, Figure 9). The microstructure and specifically the size and distribution of the pores do not facilitate cell entry into the structure. However, the cells were able to interact with the surface of the S-1200 ceramic material. The microstructure of the S-1400 promoted the formation of larger particles (Figure 7e,f) and had a complex morphology that facilitated the distribution of the cells within its structure and formation of bigger clusters between the cells and the ceramic material aggregates (Figure 9).

The microstructural differences of the ceramic materials may impact the ability of the cells for growth and proliferation. The effect of the glass-ceramic materials on cell viability of HL-60 osteoclast like cells was determined using MTT assay. The samples induced a dose-dependent decline in the cell viability (Figure 10).

The IC_50_ concentration for S-700 was 48.3 µg mL^−1^, while the IC_50_ for S-1400 was 2.5 µg mL^−1^, indicating that S-1400 had a stronger inhibitory effect on the cell viability and growth (Figure 10). The specific microstructure of the ceramic materials, the phase composition, and various Si/P ratios of C_2_S-C_3_P solid solutions might be important factors affecting their interaction with the cells and, consequently, cellular cytotoxicity. The bioceramics, based on C_2_S, are biocompatible with good osteoinductivity and integration properties [61]. While the low cytotoxicity of S-700 can be related with distinct micro-composition of the material, S-1200 and S-1400 had similarities in the phase composition (availability C_2_S-C_3_P solid solutions), but the cell viability was 10 times less after treatment with S-1200 than S-1400. In our study, solid solutions in S-1200 and S-1400 had various Si/P atomic ratios (between 1.73 and 3.59 for S-1200 and 1.67–1.84 for S-1400, respectively), proposing that the optimal Si/P ratio can be a crucial factor for the biosafety of the ceramic materials. Similarly, Yu et al. [62] have observed that the toxicity of SiO_2_ nanoparticles is cell-type dependent, and the surface charge and pore size govern the cellular toxicity. Silicocarnotite, the other major phase in C-1200, may also contribute to the difference in cell survival.

The Si/P ratio can have various impacts on cell function, as both Si and P can be released by the glass-ceramics in the cell culture. The recent systematic review of the literature has presented an analysis of the published data related to the effects of Si released from the bioceramics. The authors have commented that Si may have various effects depending on the cell type, origin, and behavior and the glass types, doping ions, and composition [63]. The negative activities for cells were related with decreased metabolic activity and biomineralization, while positive effects were associated with increased proliferation, metabolic activity, expression of angiogenic factors, extracellular matrix production, and decreased cell death [63]. Most of the data have reported more positive than negative outcomes for the cells with up/down-regulation of inflammatory factors [63]. Interestingly, the authors have shown that human cells could tolerate higher Si concentration levels released from the bioceramics than those of non-human cells [63]. We observed that Si/P ratio is important for cell adhesion, and in the long-term, ability to dissolve and release Si can impact collagen synthesis and calcification of bone tissues [64]. The release of P can stimulate matrix protein synthesis and bone formation [64].

**Figure 11:**
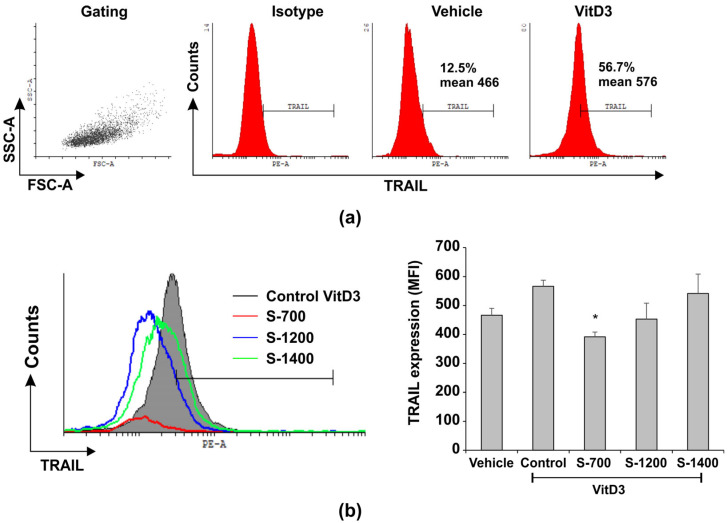
Effect of S-700, S-1200, and S-1400 on the expression of TRAIL on osteoclast-like HL-60 cells. The cells were treated with VitD3 as described in Figure 10. They were treated with the ceramic materials at concentration 5 µg mL^−1^. (**a**). Gating strategy showing the gate of cells and the expression of TRAIL on control vehicle- or VitD3-treated osteoclast-like cells. (**b**). Overlayed histogram and graph showing a down-regulation of TRAIL on VitD3-treated cells by S-700, S-1200, and S-1400. Data on the graph represent mean ± SD of three samples per group of two independent experiments (*n* = 6); * *p* < 0.05 vs. Control group, ANOVA test.

A marker for pre-osteoclasts is the TNF family member receptor activator for nuclear factor κB ligand (TRAIL). The expression of TRAIL was elevated on VitD3-treated HL-60 osteoclast-like cells (Figure 11a,b). S-700 exposure decreased TRAIL, while S-1400 inhibited cell growth and failed to inhibit TRAIL expression (Figure 11a,b).

The ability of S-700 to decrease TRAIL proposed that differentiating osteoclast-like cells might survive better. Indeed, low cytotoxicity of S-700 was determined in comparison to S-1200 and S-1400 (Figure 10). Our hypothesis is that S-700 may impact VitD3-induced osteoclast early differentiation and survival, suggesting that it is a good biocompatible material. The effect of S-1200 and S-1400 on TRAIL expression could be more complicated and may be defined by their microstructure. S-1200 and S-1400 sustained the expression of the apoptotic marker TRAIL, probably rendering osteoclast-like cells susceptible to apoptosis, but elevated TRAIL can guide TRAF6-mediated osteoclast differentiation [65]. Hence TRAIL unexpectedly can inhibit RANKL-induced osteoclast differentiation [65], showing an apoptosis-independent pathway of osteoclast differentiation and complex regulation of osteoclast functions [65]. It has been shown that the osteoinductive materials can stimulate bone regeneration. They can enhance osteogenic cytokines secretion and can drive early polarization and formation of osteogenic precursors, all dependent on the expression of receptor activator of nuclear factor kappa-Β ligand (RANKL) and TRAIL [66]. Failure of S-1200 and S-1400 to affect TRAIL expression may be related to increased apoptosis and reduced cell viability but also to driven osteoclast differentiation. S-1400 showed porous microstructure and aggregates formation, interactions with the cells, and distribution of the cells within the material, although of a smaller surface area. Thus, we speculated that the material may be used as a scaffold and may also have some osteoinductive properties despite its apoptotic action as it sustained TRAIL expression. However, this hypothesis needs further approval in the future.

## 4. Conclusions

Herein, we presented three glass-ceramic materials in the C_2_S-C_3_P system with the composition 6C_2_S.C_3_P (Ca_15_(PO_4)3_(SiO_4_)_6_) synthesized by the sol-gel method. We observed various microstructure and phase formations depending on the synthesis temperature that determined the corresponding influence on cell viability and expression of apoptotic and osteoclast-related marker TRAIL. We found that all materials were biocompatible at low concentrations for HL-60 osteoclast-like cells. We speculated that S-1400 may have application as a scaffold because of its microstructure.

## Figures and Tables

**Table 1:** Chemical composition of the glass-ceramic sample S-1200.

Oxides	Nominal Composition Ca_15_(PO_4_)_2_(SiO_4_)_6_ (Mass %)	Experimental Composition (Mass %)
CaO	62.60	59.24 ± 3.15
SiO_2_	26.83	26.32 ± 1.38
P_2_O_5_	10.56	12.78 ± 0.67
MgO	-	0.50 ± 0.06
Al_2_O_3_	-	0.10 ± 0.02
Fe_2_O_3_	-	0.06 ± 0.01
MnO	-	0.04 ± 0.01

**Table 2:** Results from the Rietveld refinement for the phase composition of the sample S-700.

Crystalline Phases	β-Ca_2_(SiO_4_)	Ca_5_(PO_4_)_3_(OH) Hydroxyapatite
Weight fraction, %	54 (2)	46 (2)
**Symmetry**		
Crystal System	Monoclinic	Hexagonal
Space group	SG *P*12_1_/*c*1 (14)	SG *P*6_3_/*m* (176)
**Unit Cell Parameters**		
*a*, Å	5.40 (3)	9.42 (4)
*b*, Å	6.77 (3)	9.42 (4)
*c*, Å	10.83 (5)	6.91 (3)
*β*, °	118.33 (8)	

**Table 3:** Results from the Rietveld refinement for the phase composition of the sample S-1200.

Crystalline Phases	Ca_15_(PO_4_)_2_(SiO_4_)_6_	Ca_5_(PO_4_)_2_(SiO_4_) Silicocarnotite
Weight fraction, %	63 (1)	21 (1)
**Symmetry** Crystal System Space group **Unit Cell Parameters** *a*, Å *b*, Å *c*, Å	Orthorhombic SG *Pnm*2_1_ (31) 21.67 (1) 9.367 (5) 6.835 (2)	Orthorhombic SG *Pnma* (62) 6.723 (3) 15.433 (6) 10.092 (4)
**Crystalline Phases**	**CaSiO_3_** **Pseudowollastonite**	**Ca_5_(PO_4_)_3_(OH)** **Hydroxyapatite**
Weight fraction, %	11 (1)	5 (1)
**Symmetry** Crystal System Space group **Unit Cell Parameters** *a*, Å *b*, Å *c*, Å *β*, °	Monoclinic SG *C*12/*c*1 (15) 11.796 (9) 6.851 (5) 10.524 (8) 111.61 (6)	Hexagonal SG *P*6_3_/*m* (176) 9.38 (1) 9.38 (1) 6.934 (9)

**Table 4:** Results from the Rietveld refinement for the phase composition of the sample S-1400.

Crystalline Phases	α-Ca_2_SiO_4_	CaSiO_3_ Pseudowollastonite	Ca_15_(PO_4_)_2_(SiO_4_)_6_
Weight fraction, %	68 (3)	21 (1)	11 (1)
**Symmetry** Crystal System Space group **Unit Cell Parameters** *a*, Å *b*, Å *c*, Å *β*, °	Hexagonal SG *P*6_3_/*mmc* (194) 5.3963 (6) 5.3963 (6) 7.090 (1)	Monoclinic SG *C*12/*c*1 (15) 6.848 (4) 11.815 (7) 19.97 (2) 90.57 (7)	Orthorhombic SG *Pnm*2_1_ (31) 21.593 (1) 9.335 (1) 7.017 (1)

**Table 5:** Adsorption-texture parameters of the synthesized glass-ceramics.

	Samples		
Parameters	S-700	S-1200	S-1400
S_BET_ (m^2^ g^−1^)	55	3	1.8
V_total_ (cm^3^ g^−1^)	0.320	0.006	0.003
D_average_ (nm)	23	7.9	6.1
V_mi_ (cm^3^ g^−1^)	0.003	-	-
S_mi_ (m^2^ g^−1^)	7	-	-
S_ext_ (m^2^ g^−1^)	48	-	-

## Data Availability

The original contributions presented in this study are included in the article/Supplementary Materials. Further inquiries can be directed to the corresponding author.

## Supplementary Materials

The following supporting information can be downloaded at: https://www.mdpi.com/article/10.3390/ma18163887/s1, Figure S1. DTA, TG and DTG curves of the dry gel. Figure S2. Samples S-1400 and S-1200. (a) Samples obtained after thermal treatment at 1400 °C (left) and 1200 °C (right). (b) Shrinkage of the powder material after thermal treatment at 1400 °C in a corundum crucible—sample S-1400. Figure S3. Optical micrographs (reflected light, Stereomicroscope Stemi 305, Zeiss). Figure S4. Optical micrographs of samples S-700, S-1200 and S-1400 in immersion liquid (transmitted light, Axioscope 5, Zeiss).
