# Supplementary material for: New Glass-Ceramics in the System Ca2SiO4-Ca3(PO4)2—Phase Composition, Microstructure, and Effect on the Cell Viability
Source: Materials (Basel). 2025 Aug 19;18(16):3887. doi: 10.3390/ma18163887 (PMC12387915; doi:10.3390/ma18163887)
Supplement: Supplementary file 1 [file materials-18-03887-s001.zip › materials-3776278-supplementary.pdf]

Supplementary figures

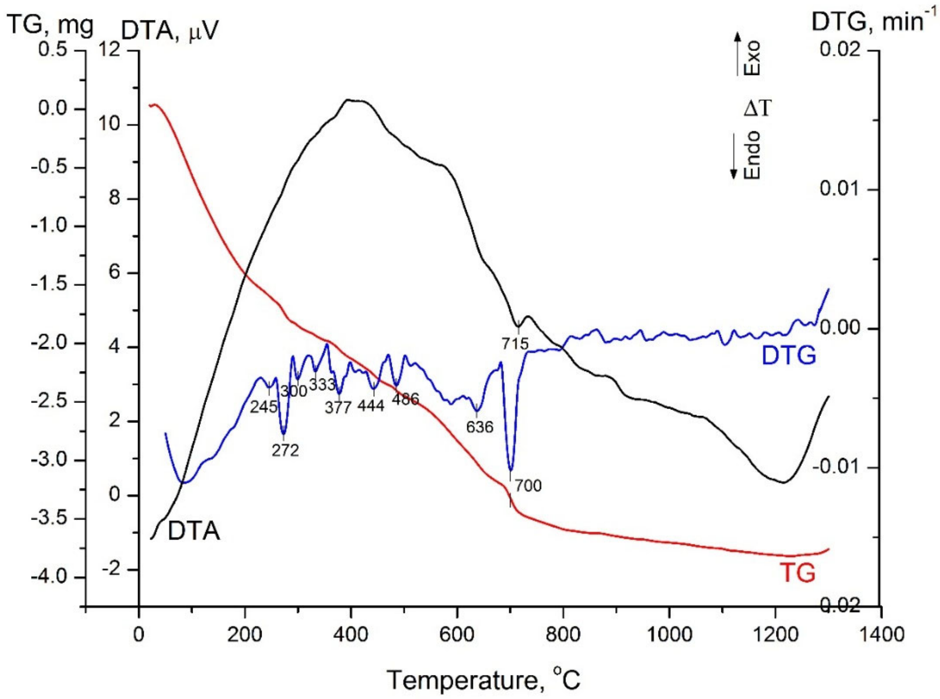

Figure S1. DTA, TG and DTG curves of the dry gel

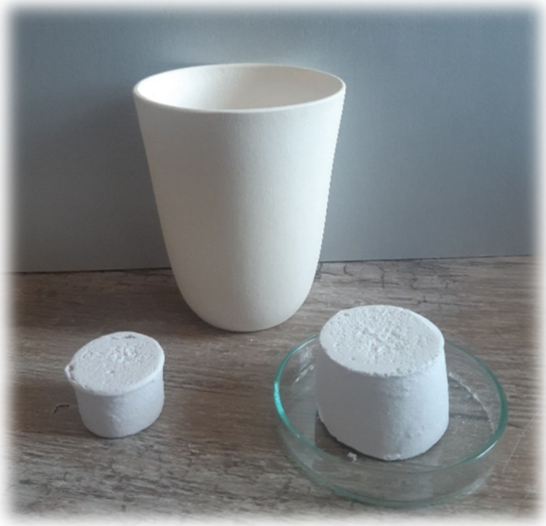

(a)

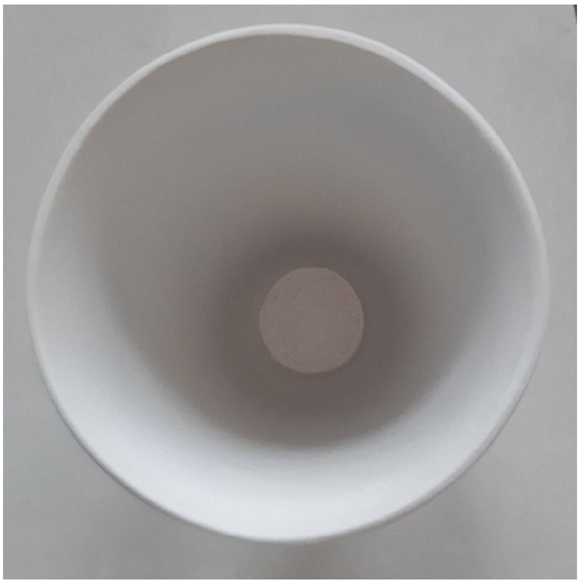

(b)

Figure S2. Samples S-1400 and S-1200. **(a)** Samples obtained after thermal treatment at 1400 °C (left) and 1200 °C (right). **(b)** Shrinkage of the powder material after thermal treatment at 1400 °C in a corundum crucible – sample S-1400.

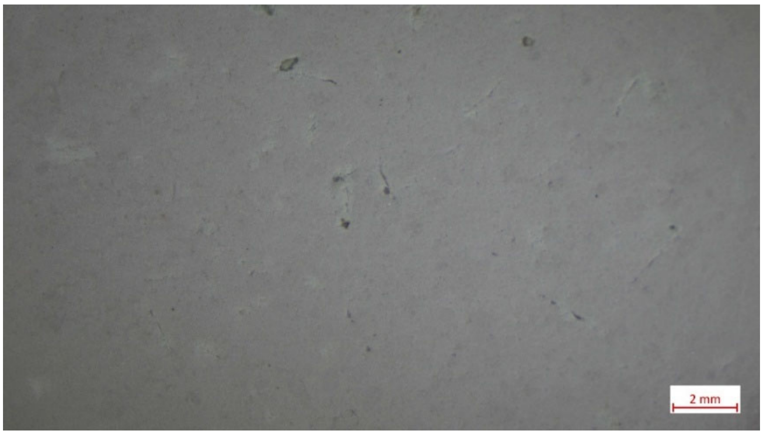

(a) Sample S-1400 - cut surface

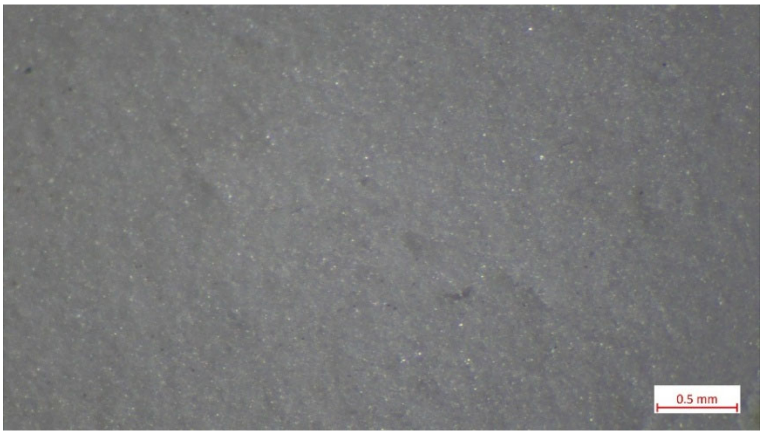

(b) Sample S-1400 - cut surface

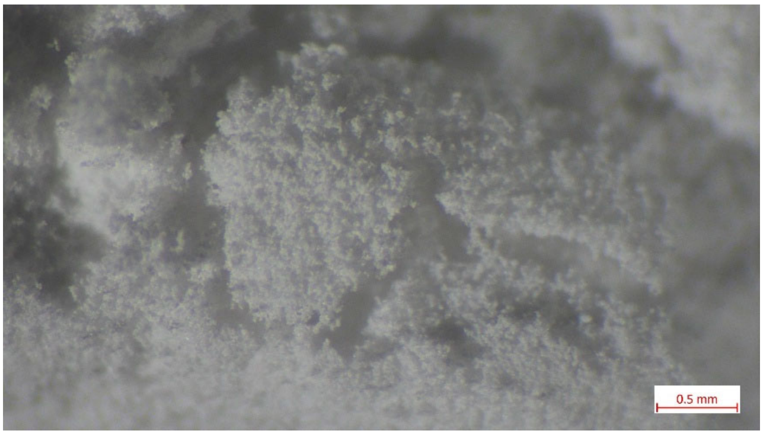

(c) Sample S-1200 - chipped surface

Figure S3. Optical micrographs (reflected light, Stereomicroscope Stemi 305, Zeiss)

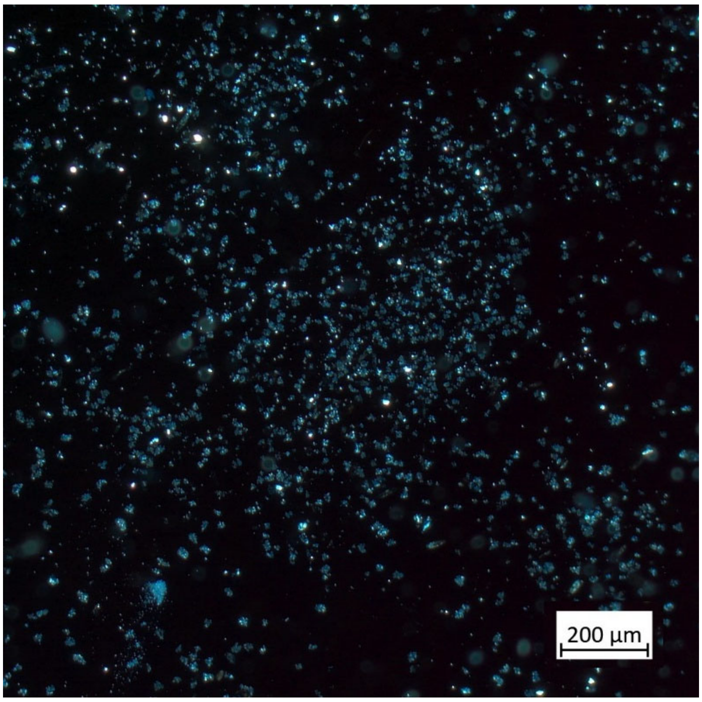

(a) S-700 cross polarized light (XPL) image

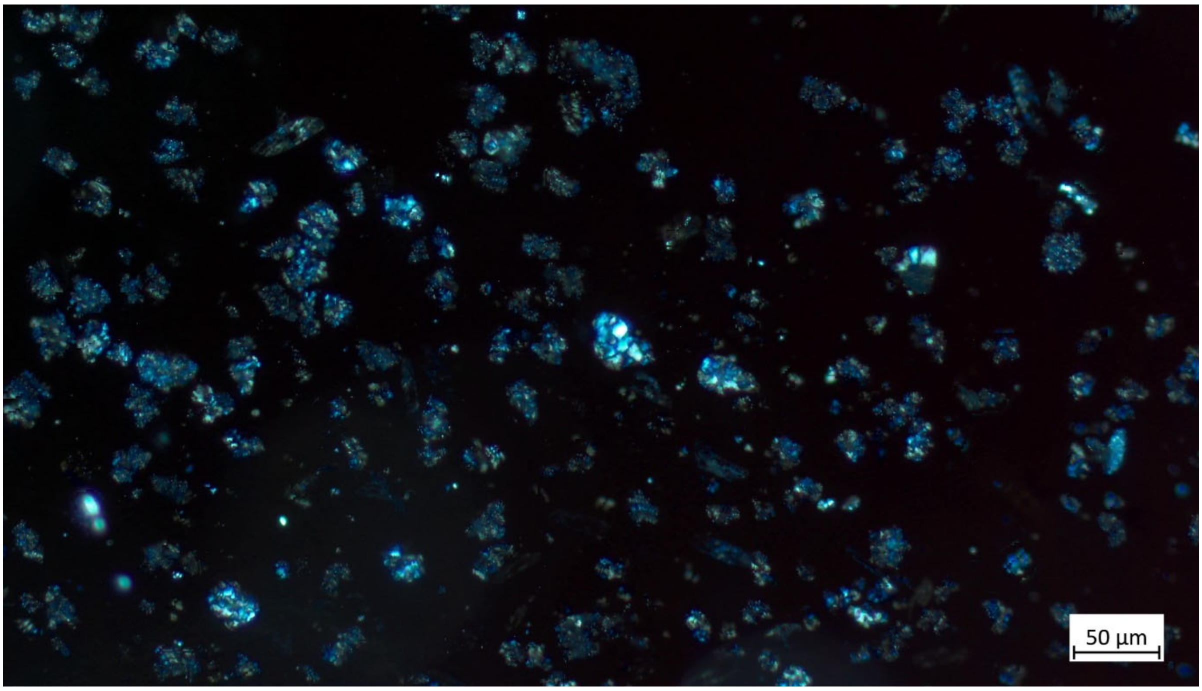

(b) S-700 XPL image

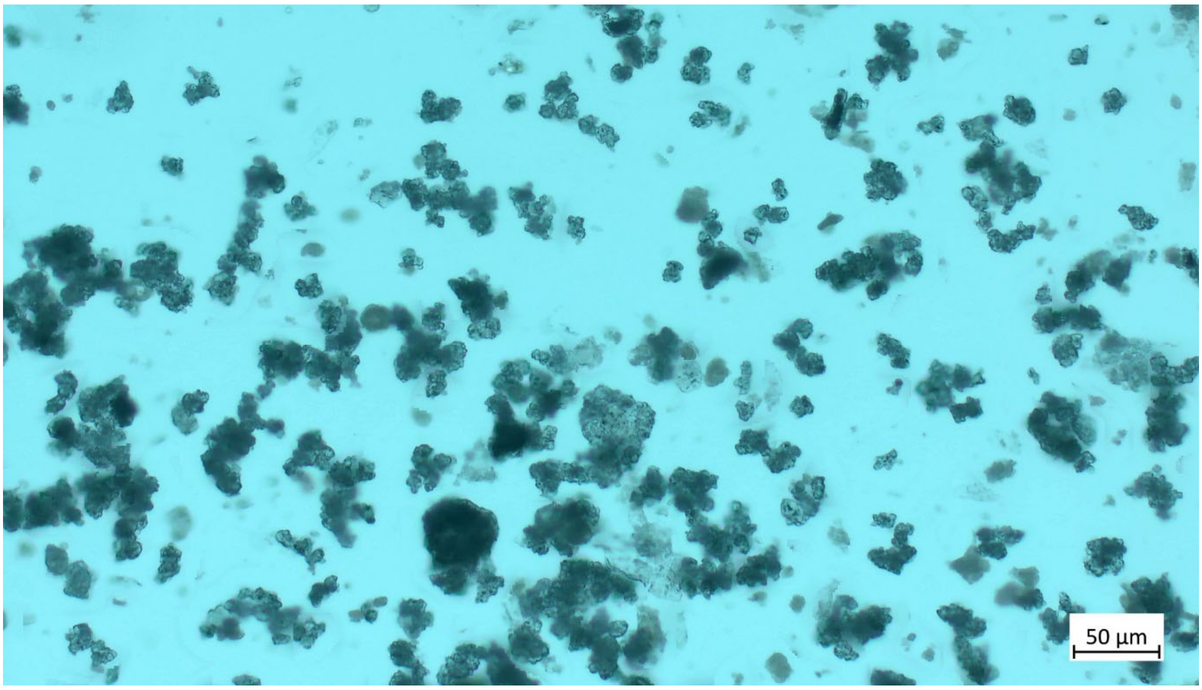

(c) S-1200 plan polarized light (PPL) image

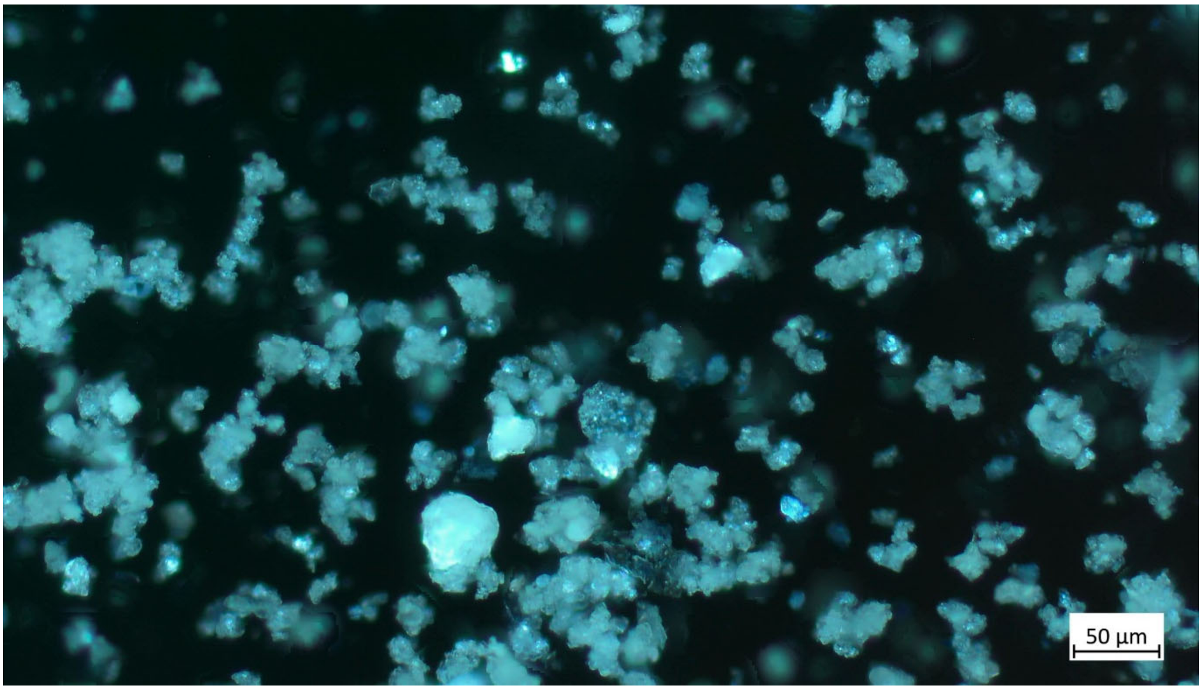

(d) S-1200 XPL image

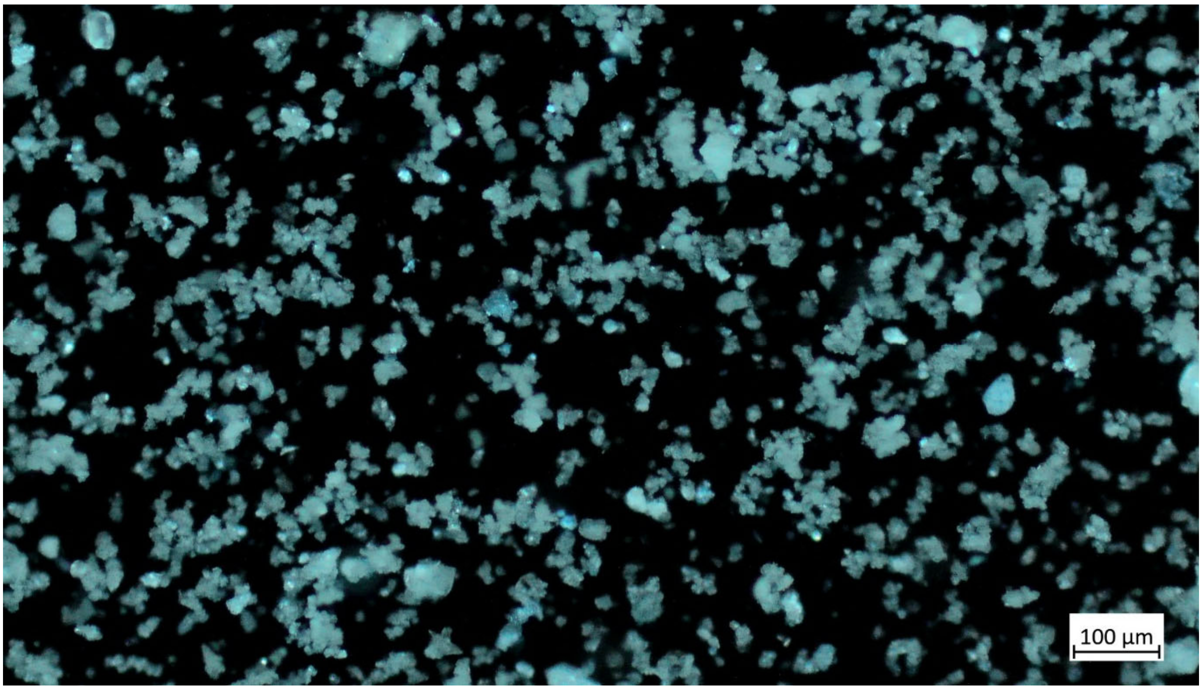

(e) S-1200 XPL image

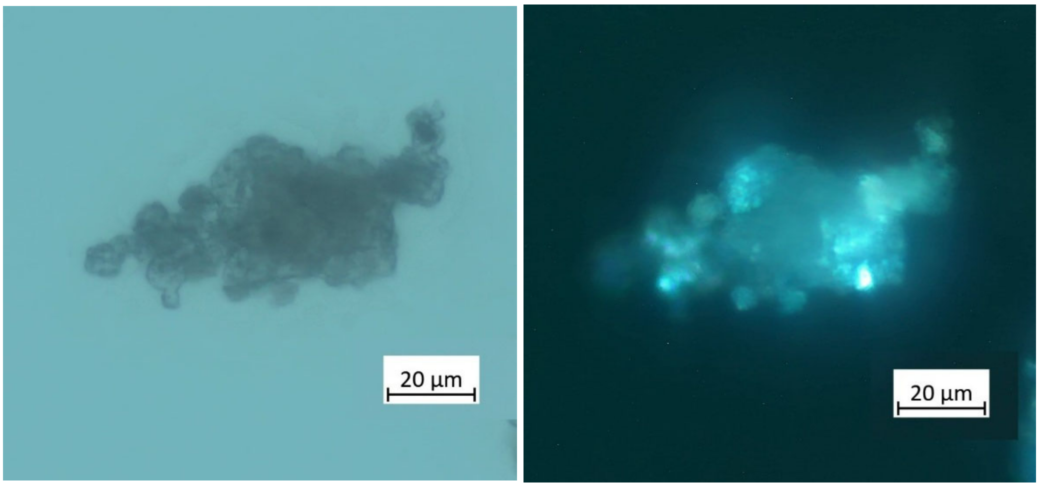

(f) S-1200 PPL and XPL images

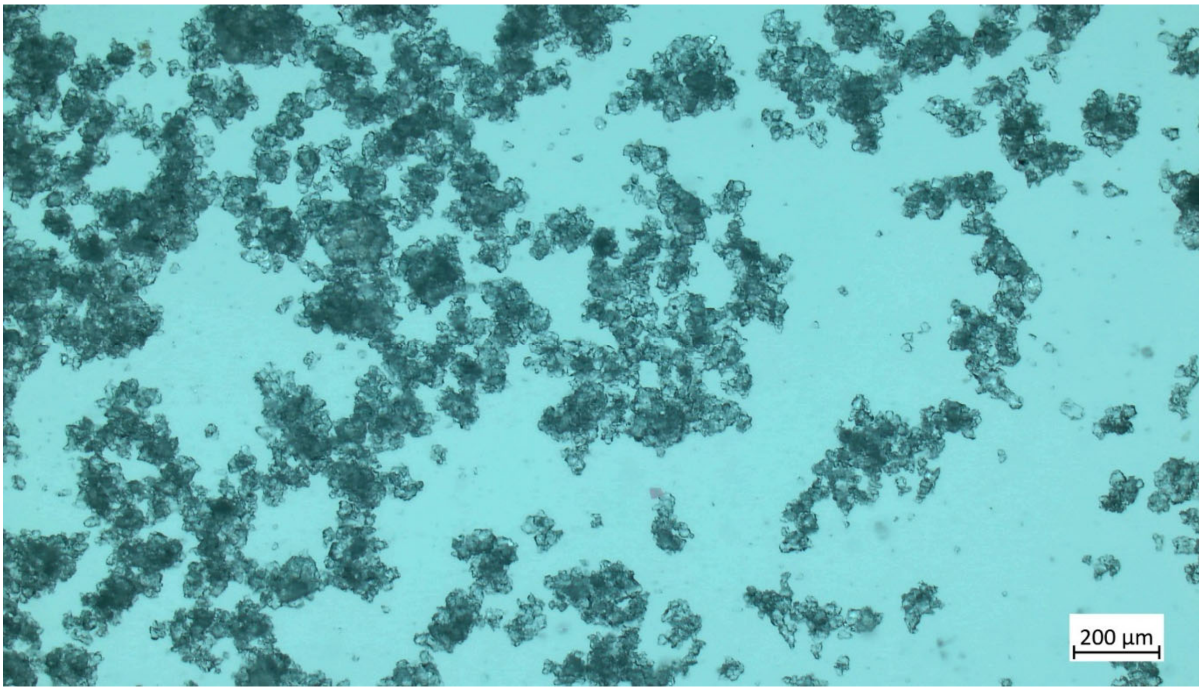

(g) S-1400 PPL image

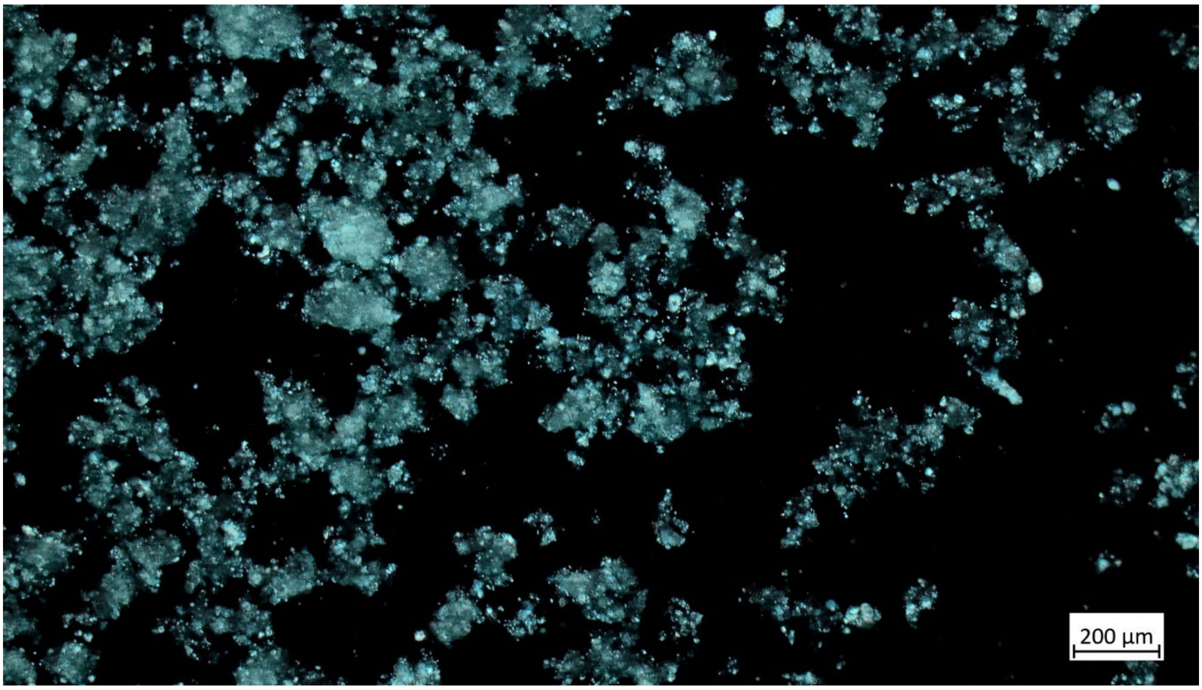

(h) S-1400 XPL image

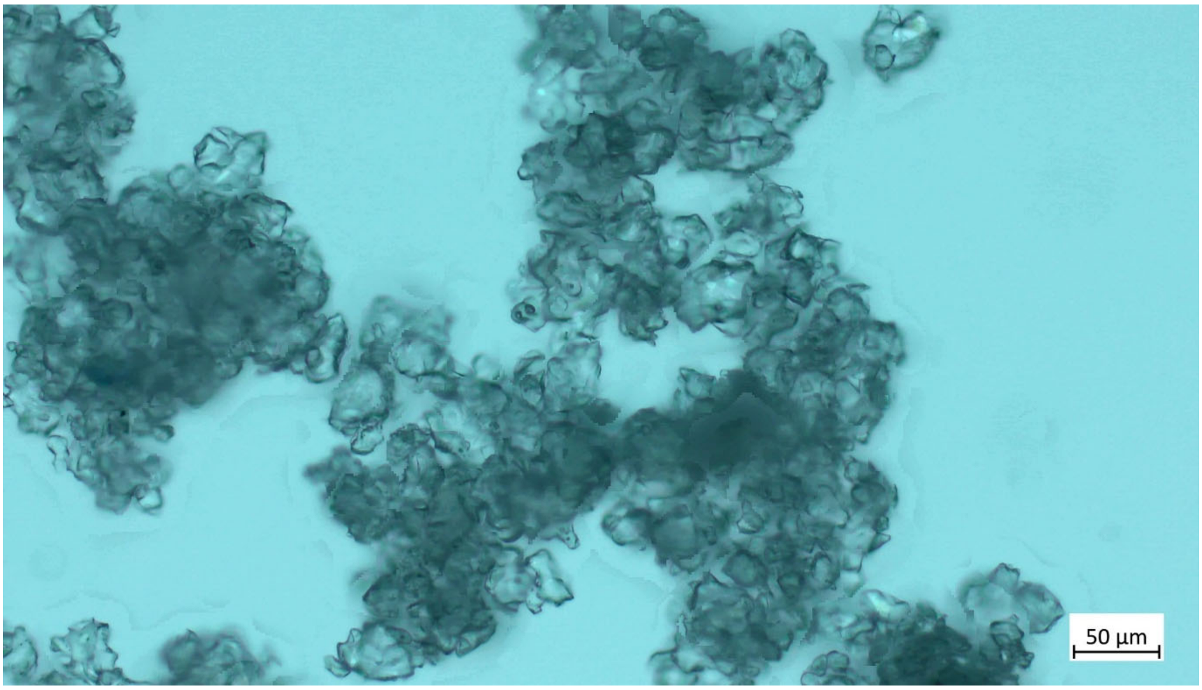

(i) S-1400 PPL image

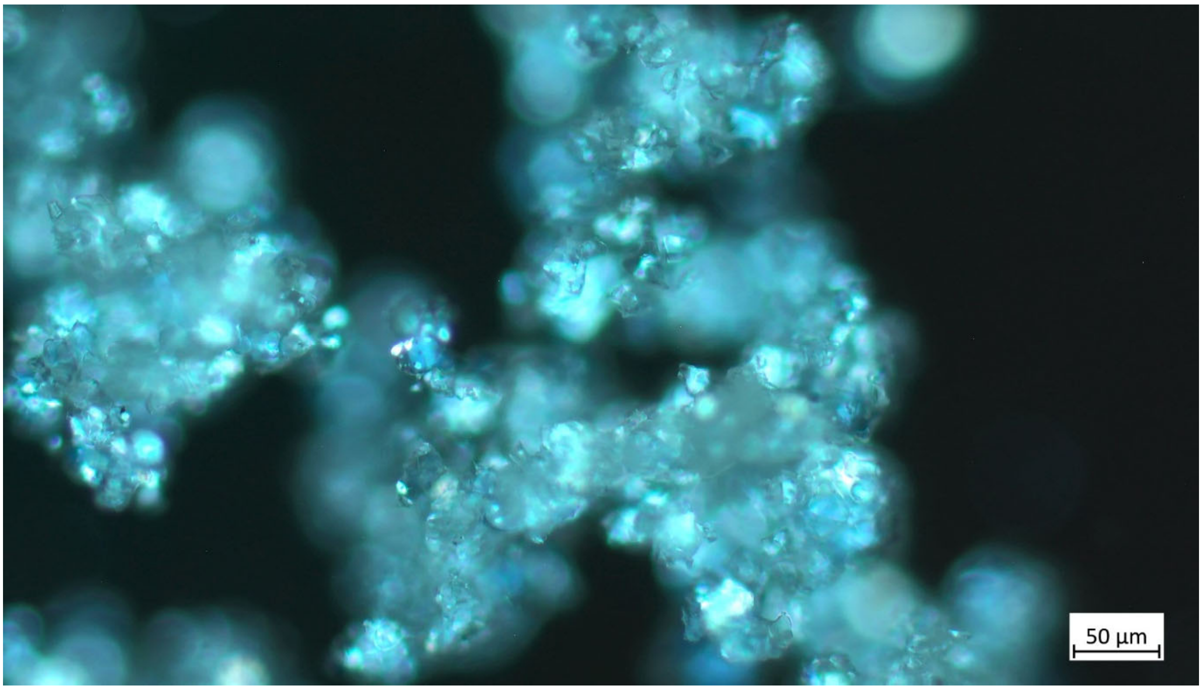

(j) S-1400 XPL image

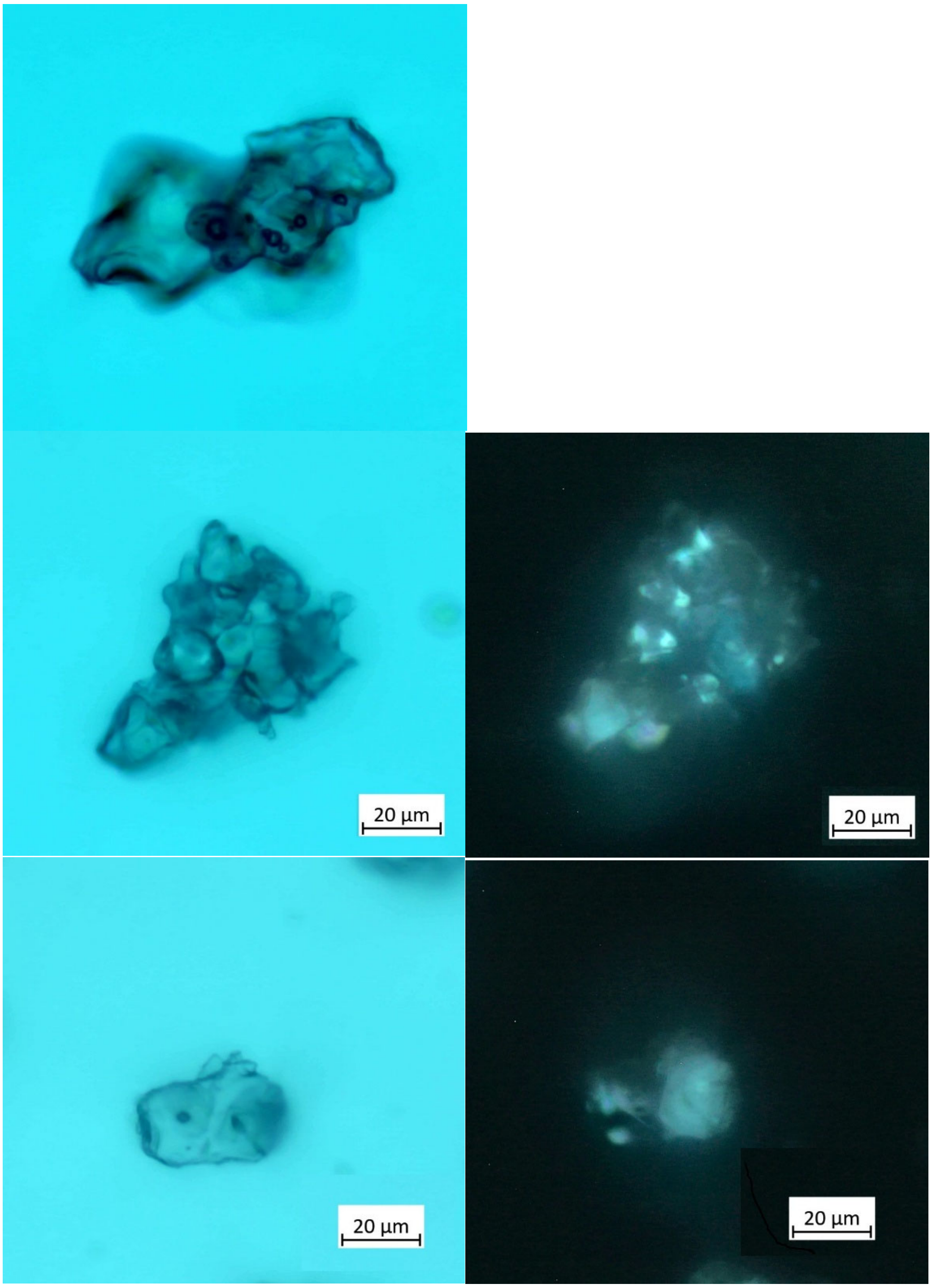

(k) S-1200 PPL and XPL images

Figure S4. Optical micrographs of samples S-700, S-1200 and S-1400 in immersion liquid (transmitted light, Axioscope 5, Zeiss)
